# Patterns of authorship in ecology and evolution: First, last, and corresponding authorship vary with gender and geography

**DOI:** 10.1002/ece3.4584

**Published:** 2018-11-08

**Authors:** Charles W. Fox, Josiah P. Ritchey, C. E. Timothy Paine

**Affiliations:** ^1^ Department of Entomology University of Kentucky Lexington Kentucky; ^2^ Ecosystem Management, School of Environmental and Rural Science University of New England Armidale NSW Australia

**Keywords:** authorship, gender bias, gender discrimination, women in science

## Abstract

The position of an author on the byline of a paper affects the inferences readers make about their contributions to the research. We examine gender differences in authorship in the ecology literature using two datasets: submissions to six journals between 2010 and 2015 (regardless of whether they were accepted), and manuscripts published by 151 journals between 2009 and 2015. Women were less likely to be last (i.e., “senior”) authors (averaging ~23% across journals, years, and datasets) and sole authors (~24%), but more likely to be first author (~38%), relative to their overall frequency of authorship (~31%). However, the proportion of women in all authorship roles, except sole authorship, has increased year‐on‐year. Women were less likely to be authors on papers with male last authors, and all‐male papers were more abundant than expected given the overall gender ratio. Women were equally well represented on papers published in higher versus lower impact factor journals at all authorship positions. Female first authors were less likely to serve as corresponding author of their papers; this difference increased with the degree of gender inequality in the author's home country, but did not depend on the gender of the last author. First authors from non‐English‐speaking countries were less likely to serve as corresponding author of their papers, especially if the last author was from an English‐speaking country. That women more often delegate corresponding authorship to one of their coauthors may increase the likelihood that readers undervalue their role in the research by shifting credit for their contributions to coauthors. We suggest that author contribution statements be more universally adopted and that these statements declare how and/or why the corresponding author was selected for this role.

## 1 INTRODUCTION

Publishing scholarly articles is the primary means by which scientific research is communicated. Being an author on a peer‐reviewed scholarly publication is thus the predominant means by which researchers get credit for their research contributions when applying for jobs and promotion (Wren et al., 2007), and for grants. When manuscripts have more than one author, the contributions of authors to the research can vary substantially among individuals, both in type (e.g., conceptualization, data collection, data analysis) and in magnitude. The extent of each author's contribution can be inferred from the order in which their names appear in the byline (Logan, Bean, & Myers, 2017), though the conventional meanings of authorship positions vary among research disciplines and countries (Liu & Fang, 2014; Waltman, 2012). The most common convention is for the first author to be the person who contributed the most to a project and the last author to be the person who supervised the project (Baerlocher, Newton, Gautam, Tomlinson, & Detsky, 2007; Corrêa, Silva, Costa, & Amancio, 2017; Costas & Bordons, 2011; Larivière et al., 2016; Marušić, Bošnjak, & Jerončić, 2011; Perneger et al., 2017; Sundling, 2017; Yang, Wolfram, & Wang, 2017), though there are necessarily many exceptions to this convention, especially when coauthors are of equivalent professional rank rather than in a mentee–mentor relationship. Surveys indicate that ecologists tend to assume that the first author contributed the most time and energy to the project (Weltzin, Belote, Williams, Keller, & Engel, 2006) and that the last author is the senior researcher (e.g., head of laboratory) under whose guidance the research was done (Duffy, 2017). Similar assumptions are common across the biological sciences (Wren et al., 2007; Zbar & Frank, 2011), though authorship conventions differ in other fields (Costas & Bordons, 2011; Marušić et al., 2011). Also, as the number of authors on manuscripts has increased over time (e.g., Fox, Paine, & Sauterey, 2016, Logan, 2016, Duffy, 2017 for ecology), the number of middle authors, and their collective contribution to the research, has necessarily increased (Mongeon, Smith, Joyal, & Larivière, 2017). Given that the position of an author on the byline of a paper affects reader's assessments of their contributions, variation between men and women or among cultures in authorship roles can affect career success.

Most published manuscripts list one or rarely two individuals to whom correspondence about the manuscript should be addressed. Survey studies have found that the person who takes responsibility for manuscript correspondence is generally assumed to have led study conception, design, and publication, regardless of their position in the author order (Bhandari et al., 2004, 2014 ). Thus, authorship credit schemas used in the infometrics literature commonly assign substantial authorship credit to the corresponding author (Xu, Ding, Song, & Chambers, 2016). Most often the first author is the corresponding author, though this varies among countries and journals, with last author being the next most common corresponding author (Duffy, 2017; Matteson, Sundberg, & Laget, 2011). However, when the first author is not the corresponding author, readers commonly assume that the corresponding author deserves credit for study conception, design, analysis, and interpretation, reducing their perception of the first author's role (Bhandari et al., 2014; Bhandari, Einhorn, Swiontkowski, & Heckman, 2003; Wren et al., 2007). Thus, differences in corresponding authorship practices between men and women or among cultures, can also affect the credit authors receive for their research contributions.

It has been widely demonstrated that the representation of women as authors on scholarly publications varies according to their authorship role, with men historically dominating the first and last author positions on manuscripts (West, Jacquet, King, Correll, & Bergstrom, 2013). However, the representation of women has gradually been increasing, with women now generally well represented in many fields at the first author position, but still underrepresented as last (i.e., “senior”) authors, relative to their representation at other authorship positions (e.g., biomedical literature, Oertelt‐Prigione, 2012; ecology, West et al., 2013). Less is known about gender differences in corresponding authorship. In one recent study of an ecology journal, Fox, Burns, Muncy, and Meyer (2016) found that women were 8% less likely than men to serve as corresponding author when they were first author on a manuscript, a pattern also found in some biomedical journals (Heckenberg & Druml, 2010; Yun et al., 2015). However, the generality of these observations is unknown.

The objective of this study is to examine gender differences in authorship patterns in the ecological literature using two datasets. Our first dataset includes all research manuscripts submitted to six journals in ecology and evolution between 2010 and 2015, regardless of final disposition (accepted or rejected) of the manuscript. Our second dataset includes all research manuscripts published by 152 ecology journals between 2009 and 2015. In addition to describing the representation of women as first and senior authors in ecology, we examine how the representation of women varies among journals, with the gender of their coauthors and with the impact factor of the journal in which they publish. We also examine differences in corresponding authorship practices between men and women, and how these practices vary geographically (e.g., whether they vary with global indices of gender inequality). Specifically, we examine (a) gender differences in authorship and corresponding authorship, how they have varied over time, and how they vary depending upon the geographic location of the authors. We test (b) how gender differences in authorship vary with journal prominence, (c) whether the proportion of women on a paper varies with the gender of the last author, (d) whether the distribution of genders on multiauthored papers deviates from random expectation given the overall observed proportion of male and female authors, and (e) whether papers with female first or last authors have more or fewer authors than do papers authored by men.

## 2 METHODS

### 2.1 The datasets

#### 2.1.1 Submitted papers

We extracted all metadata and peer review details for all manuscripts submitted to six ecology and evolution journals from *ScholarOne Manuscripts. *We included manuscripts submitted between 1 January 2010 and 30 June 2015 for *Functional Ecology*, *J Animal Ecology*, *J Applied Ecology*, *J Ecology*, and *Methods in Ecology and Evolution* (this journal received its first ever submission on 13 August 2009), and between 1 January 2010 and 31 December 2015 for *Evolution.* The dataset includes only standard research papers (called a “Research Article” at *Methods in Ecol Evol*, an “Original Article” at *Evolution*, and a “Standard Paper” at the other journals). We consider only the first submission of a manuscript; revisions and resubmissions were excluded (so that we do not double count papers). Data in ScholarOne are author‐entered and so author lists in the database are sometimes incomplete and often incorrectly ordered. We thus determined authorship order and corresponding authorship on papers from the cover page of the submitted manuscript. The dataset includes 23,713 manuscripts.

#### 2.1.2 Published papers

We extracted metadata for all manuscripts in the ecology domain published from 2009 to 2015 from *Clarivate Analytics *Web of Science. Review journals such as *Trends in Ecology and Evolution* and the *Annual Review of Ecology, Evolution, and Systematics* were excluded, as most papers in those journals are invited. We also excluded review papers, commentaries, perspectives, editorials, brief communications, and other types of papers not considered typical full‐length research studies. The corresponding author was the listed reprint author in Web of Science. This dataset includes 152 journals and 95,589 studies for which at least one author could be genderized.

### 2.2 Author gender

For both datasets, author binary gender was determined using the online database https://genderize.io. This database includes >200,000 distinct names and the probability that each name is male or female given the distribution of genders for these names in the database. If an author's name was not listed in genderize.io or was listed but had less than a 95% probability of being one gender, we used an Internet search to determine gender. To do so, we searched for individual web pages or entries in online databases that included a photograph of the individual or other information indicating their gender. In the dataset of published papers, 16.4% of authors were listed only with initials and could not be assigned to a gender. We were able to genderize ~98% of all authors in the submitted manuscripts dataset (98.4% of first authors and 98.0% of last authors) and 77.7% of authors in the published papers dataset (79.2% of first authors and 80.3% of last authors).

### 2.3 Author geography

Our dataset contains the geographic location (country) of most authors (from author‐submitted addresses). The attribution of authors to countries was based solely upon the location of the institution with which they were affiliated, rather than the land of their birth. In the published papers dataset, for authors with multiple institutional affiliations located in multiple countries, we chose one country at random. To categorize localities, we used the M.49 area codes and their continental regions as defined by the United Nations’ Statistical Commission (unstats.un.org). There were three exceptions: (a) we divided the Americas into the two UN‐designated subareas, Latin America (which includes North America south of the United States–Mexico border; the countries in M.49 area 419) and North America (the United States, Canada, Bermuda, and Greenland; M.49 area 021), (b) we split Australia and New Zealand (M.49 area 053) off from the rest of Oceania, and (c) we divided Europe into the United Kingdom and “other Europe.” This third exception reflects the large number of papers received from the United Kingdom, and that a British learned society (the British Ecological Society) owns five of the six journals in our submitted papers dataset. The number of papers from countries in Oceania (excluding Australia & New Zealand) was very low so papers from this region were not included in analyses of geographic variation, but were included for all other analyses.

To test for the influence of gender inequality on geographic variation in the proportion of women serving in different authorship roles, we obtained two metrics of gender inequality. First, we used the 2015 Gender Inequality Index (GII) from the United Nations Development Programme (2016) (extracted on 13 November 2017). The Gender Inequality Index is a measure of gender disparity that attempts to quantify the degree to which women lose opportunities relative to men and includes estimates of gender disparity in health, empowerment, and participation in the labor force. The Gender Inequality Index is an imperfect metric of inequality (Permanyer, 2013) but is highly correlated with degree of religiosity (Klingorová & Havlíček, 2015) and various metrics of women's empowerment (Sundström, Paxton, Wang, & Lindberg, 2017). Second, we used the 2015 V‐Dem Women Civil Liberties Index (WCLI; extracted on 14 Nov 2017), which quantifies the degree to which women can make meaningful decisions in their lives (Sundström et al., 2017; data from Coppedge et al., 2017). WCLI was squared to improve its distribution for data analysis.

We have no mechanism to identify an author's proficiency in English, the written language in which the studied journals publish. The best metric we have of language proficiency is the native language of the author's country of residence. Specifically, we categorized an author as being fluent in English if English is the most common and/or an official language of their country of residence (as in Clavero, 2011). Whether English is the most common or an official language was determined from the online version of the CIA World Factbook (The World Factbook 2017, extracted on 14 November 2017). This metric is imperfect because many authors are bilingual (including English) from childhood, many who learn English as a second language are excellent at writing in English, and many researchers move among countries of different languages and thus may reside in a country at the time of manuscript submission that does not correctly indicate their native language.

### 2.4 Journal impact factors

To test for variation in gender representation among journals of different profile levels, we used journal impact factors obtained from *Clarivate Analytics *Journal Citation Reports. Because manuscripts are typically submitted to a journal one or 2 years before their eventual publication, we used journal impact factors for annual period that was 2 years prior to the publication year of the focal manuscript as our measure of journal rank at the time of manuscript submission. These impact factors are typically made public half‐way through the following year and thus would be the most recently available impact factors an author could consider when submitting their manuscript. Impact factors were log‐transformed to reduce heteroscedasticity.

### 2.5 Statistical analyses

For statistical analyses, each manuscript represents a single data point that includes one first author, one corresponding author, one author sex ratio, and so on. Many of the variables examined here are binary; for example, author gender [F/M], corresponding author [yes/no]. These variables were thus analyzed using logistic regression (SAS v9.4, Proc Logistic with link = logit) with models of the form *DependentVariable *= Year* *+ *Journal* + IndependentVariables* *+ *TwoWayInteractions*. All independent variables were treated as categorical except (a) *Year *was treated as a continuous variable (because we are interested in directional trends over time rather than simply among‐year variation), and (b) *Journal* was treated as a random effect for the published papers dataset (for which we did not test for journal*year interactions). The number of authors was cube root transformed for analysis. Further details are described as necessary as results are presented.

## 3 RESULTS

### 3.1 Patterns of authorship

**Figure 1:**
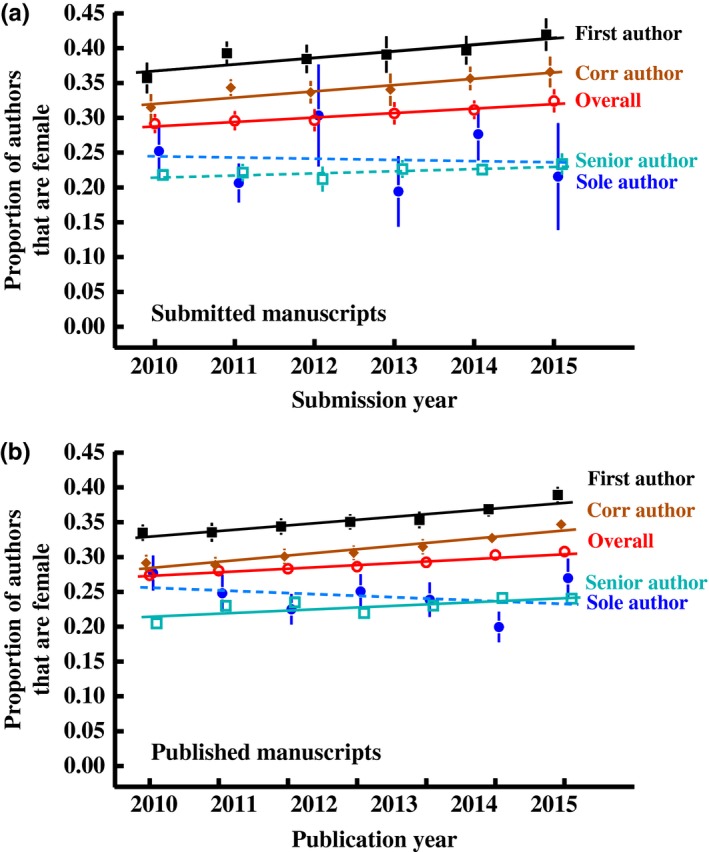
Variation among years in the proportion of authors that are female for different positions on the author list. The proportion of women overall, and in the first and senior positions, are for multiauthored papers. Sole authorship is single‐author papers. Corresponding author is the person identified on the cover page as the author to whom correspondence should be addressed. Solid lines indicate statistically significant increases over time. Means are calculated first by averaging across papers within each year, and then across journals within each year. Standard errors are calculated from the among‐journal standard deviation and are sometimes smaller than the points. Statistical models for a^1^ and b^2^ are below. ^1^Variation over time of the gender ratio of authors on submitted manuscripts: *AuthorGender *= *Journal* + *Year *+ Journal*Year interaction, with journal as a random effect and year as a covariate. First author (χ^2^_1_ = 17.5, *p* < 0.001), last author (*Year*: χ^2^_1_ = 3.28, *p* = 0.07), sole author (*Year*: χ^2^_1_ = 0.01, *p* = 0.92), overall authorship (*Year*: *F*_1,22566_ = 30.7, *p* < 0.001), corresponding author (*Year*: χ^2^_1_ = 15.2, *p* < 0.001). ^2^Variation over time of the sex ratio of authors on published manuscripts: *AuthorGender *= *Journal* + *Year*, with journal as a random effect and year as a covariate. First author (*Year*: χ^2^_1_ = 86.3, *p* < 0.001), last author (*Year*: χ^2^_1_ = 44.6, *p* < 0.001), sole author (*Year*: χ^2^_1_ = 0.47, *p* = 0.51), overall authorship (*Year*: *F*_1,__89436_ = 166.4, *p* < 0.001), corresponding author (*Year*: χ^2^_1_ = 95.3, *p* < 0.001)

**Figure A1:**
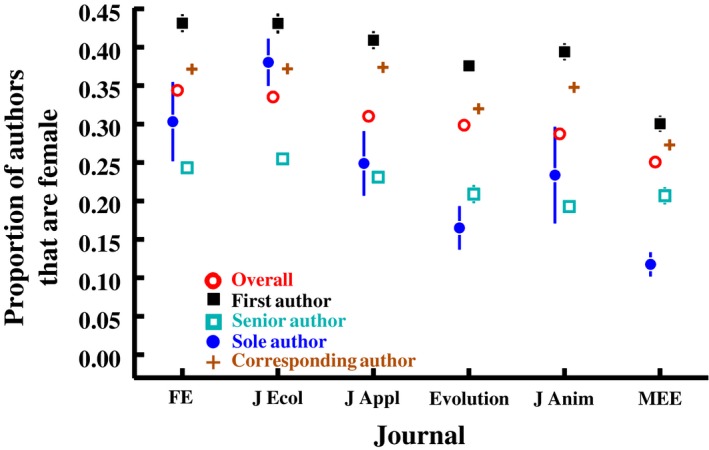
Variation among journals in the proportion of authors that are female on submitted papers for different positions on the author byline. Journals are sorted (left to right) by the overall proportion of female authors. The proportion of women overall, and in the first and senior positions, are for multiauthored papers. Sole authorship is single‐author papers. Corresponding author is the person identified on the cover page as the author to whom correspondence should be addressed. Means are calculated first by averaging across papers within each year, and then across years within each journal. Standard errors are calculated from the among‐year standard deviation and are sometimes smaller than the points

**Figure A2:**
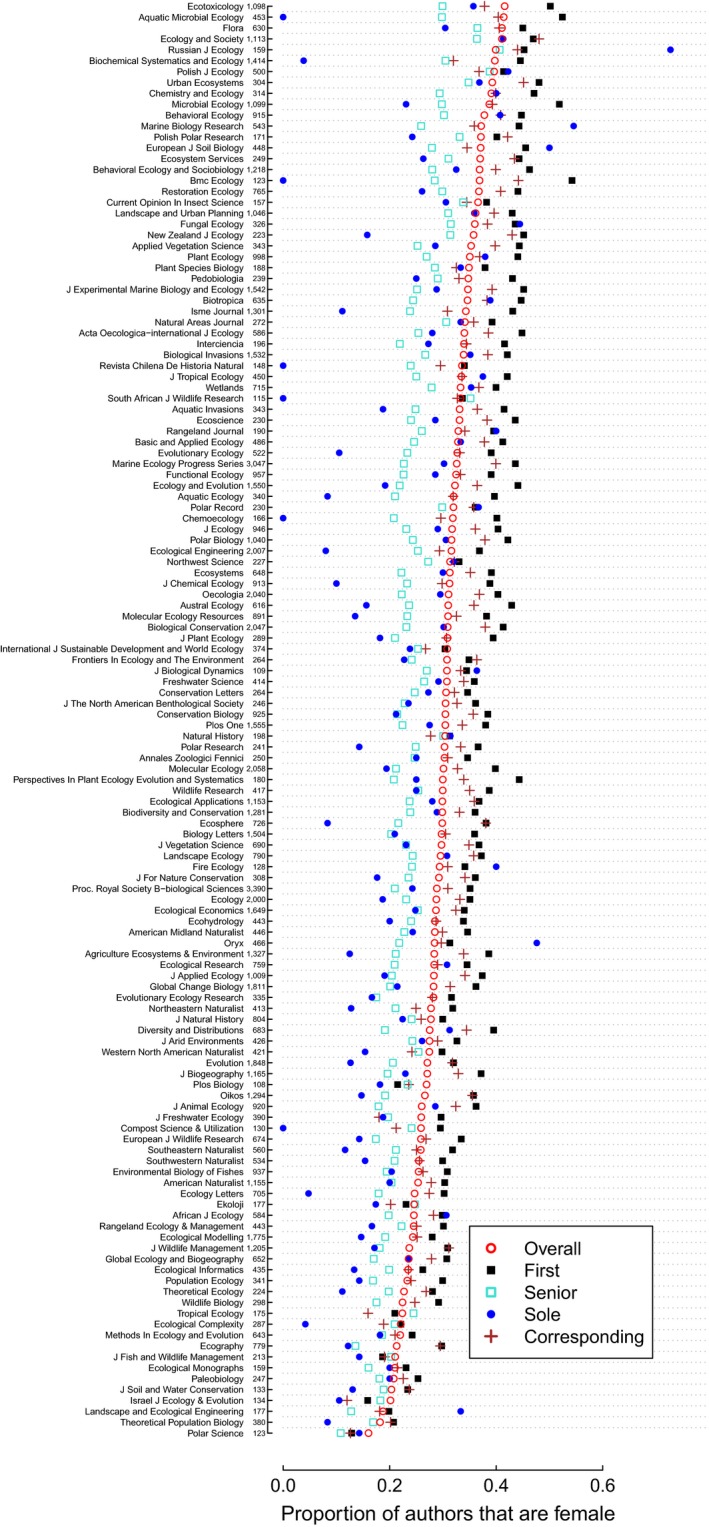
Variation among journals in the proportion of authors that are female on published papers for different positions on the author list. Journals are sorted by the overall proportion of female authors. Details as in Figure A1. Standard errors are omitted for clarity of presentation. Numbers next to each journal name represent sample sizes for each journal. The figure includes only the 132 journals for which there were at least 100 papers with at least one author genderized

Averaged across years within journals, and then across journals, women accounted for 30.4 ± *SEM* 0.5% of all authorships on multiauthored papers in the submitted papers dataset (Figure 1a) and 28.9% ± 0.5% in the published papers dataset (Figure 1b; see also Figures A1, A2). Women were more highly represented as first authors (39.0 ± 0.9 and 35.3% ± 0.8%), but less frequent as senior authors (22.3 ± 1.1 and 22.9% ± 0.5%), than expected from the overall average proportion of women across all positions (Figure 1a and 1b, respectively). Women were also substantially underrepresented as sole authors (24.1 ± 4.2 and 24.4% ± 1.0%) relative to their representation on multiauthored papers.

The overall proportion of female authors increased over the six years for which we have data (Figure 1). The average proportion of female authors on multiauthored papers increased slightly but consistently year‐on‐year from 29.1 ± 1.3 to 32.4% ± 1.6% from 2010 to 2015 in the submitted papers dataset, and from 28.0 ± 0.7 to 30.8% ± 0.7% for the published papers dataset over this same period (Figure 1). Though only about a 3 percentage point increase, this represents a *relative *increase of 11.2% and 10.0%, respectively, over just 6 years in the proportion of women among authors across all authorship positions. The proportion of women at the first author position also increased slowly but consistently over this timeframe from 35.7 ± 2.1 to 41.9% ± 2.3% in submitted papers, and from 33.5 ± 1.1 to 38.9% ± 1.3% over this same period for published papers, relative increases of 12% and 16% between 2010 and 2015 (Figure 1). There was no statistically significant increase in the proportion of women at the senior author position in the submitted papers dataset (Figure 1a), but there was a significant increase observed in the larger published papers dataset, albeit only a small relative increase 7.3%, much less than that observed for first authors (Figure 1b). There was no consistent increase in the proportion of women on single‐author manuscripts, possibly due to the relatively low number of single‐authored papers (only 5,629 out of 95,589 [5.9%] in the published papers dataset; Figure 1).

**Figure 2:**
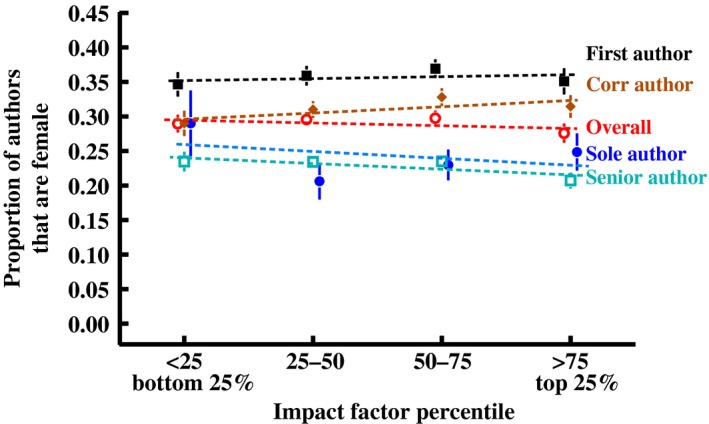
The proportion of women among authors on papers varies with journal impact factor. The frequency of women among all author positions^1^ (solid red line) declined with increasing journal impact factor, but the frequency of women as first, last, and corresponding authors (dashed lines) does not^2^. We present means (± *SEM*) per journal impact factor quartile for clarity, but individual impact factors are used in the data analysis^1,2^. Means were calculated by averaging across years within journals, then across journals within quartiles. ^1^Model for overall authorship: multiple regression on mean author sex ratio per journal per year, such that each journal contributes only one data point per year: *AuthorGenderRatio* = *Year* + *JournalImpactFactor*, with journal impact factor (JIF) log‐transformed (log[JIF+1]); *JIF*: *F*_1,844_ = 1.53, *p* = 0.22. ^2^Logistic regression with each point weighted by the inverse of the number of papers in the publishing journal that year, with journal impact factor (JIF) log‐transformed (log[JIF+1]); *NumberOfWomen/NumberOfPapers* = *Year* + *JournalImpactFactor*; JIF effects: First author (χ^2^_1_ = 0.10 *p* = 0.75), senior author (χ^2^_1_ = 0.56, *p* = 0.45), corresponding author (χ^2^_1_ = 0.24, *p* = 0.63), sole author (χ^2^_1_ = 3.03, *p* = 0.07)

The overall proportion of female authors (averaged across all author positions) did not vary significantly with journal impact factor. Similarly, there was no variation across journal impact factors in the proportion of female authors for any specific author position (first, corresponding, senior or sole author roles; Figure 2). For example, women were equally well represented as first authors at the top 25% impact factor journals (38.3% ± 2.2%) and bottom 25% impact factor journals (38.7% ± 1.9%; details and statistics in Figure 2).

### 3.2 Mixing of genders in multiauthored papers

**Figure 3:**
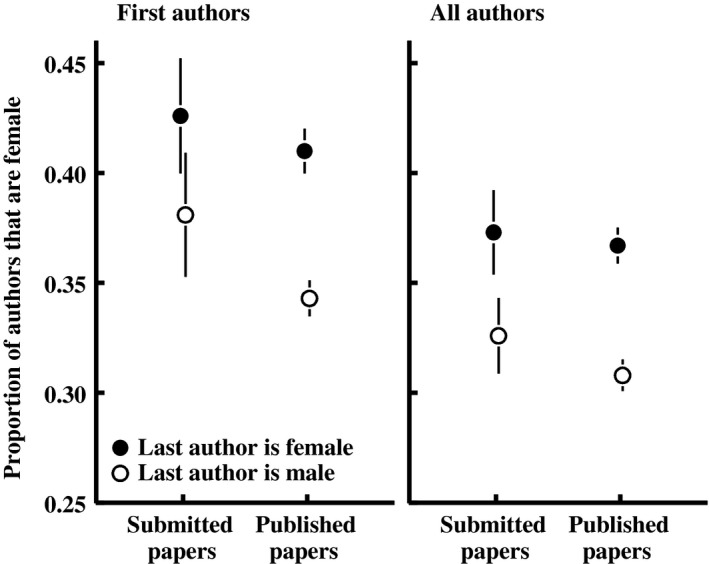
Women are more frequently authors on papers for which the senior author is female. “First author” is the proportion of first authors that are female. “All authors” is the proportion of all authors (excluding the senior) that are female. Solid points are for female senior authors, open points are male senior authors. Points are means (averaged across papers within journal*year combinations, then across years within journals, then across journals) ± *SEM*. Includes only multiauthor papers

The first author on a paper was more likely to be female if the last author was also female rather than male in both the submitted and published datasets (“First author” in Figure 3), a relative difference of 11.8% and 17.1%, respectively, in the proportion of women on papers with female versus male senior authors (*χ* ^2^_1_ = 34.6, *p* < 0.001 and χ^2^_1_ = 176.3, *p* < 0.001 for the submitted and published paper datasets, respectively). More generally, the proportion of female coauthors at all positions was higher on manuscripts with female senior authors (“All authors” in Figure 3), a *relative* difference of 14.4% and 16.1% in the representation of women authors (average across all positions excluding the senior author) on papers with female versus male senior authors (*F*_22131_ = 77.5, *p* < 0.001 and *F*_1,83489_ = 414.1, *p* < 0.001).

**Figure 4:**
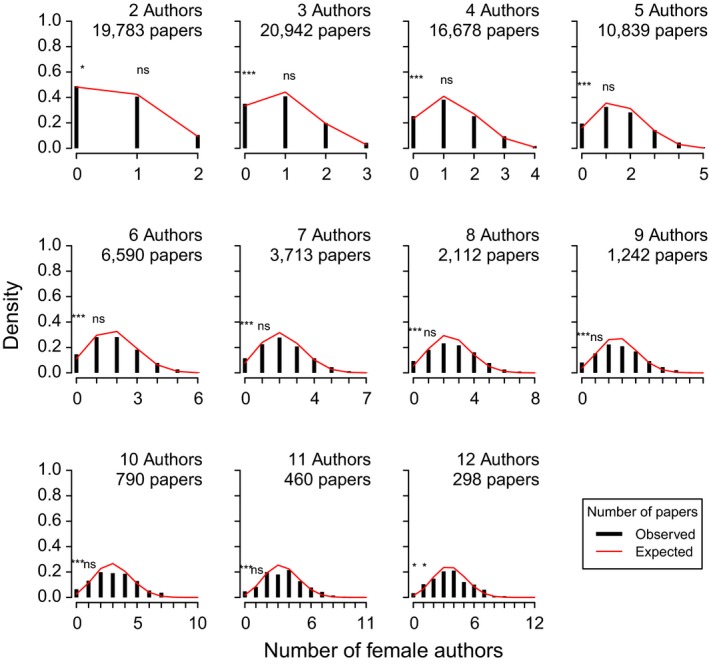
The observed distribution of papers with *N* female authors (black bars) compared to the expected distribution of papers with *N* female authors (red line) for the published papers dataset (see Figure S3 for the analogous relationships in the submitted papers dataset). The expectation is derived from a binomial distribution with the same mean as the observed average proportion of female authorship across all author positions, given *N* authors of known gender. As indicated by the stars over the left‐most bars, there was a significant overabundance of all‐male papers in all collaborations of three or more authors (*p* < 0.0001). There was no support for the “token female” hypothesis, as the number of papers with a single female author did not differ from that expected by the binomial distribution

**Figure A3:**
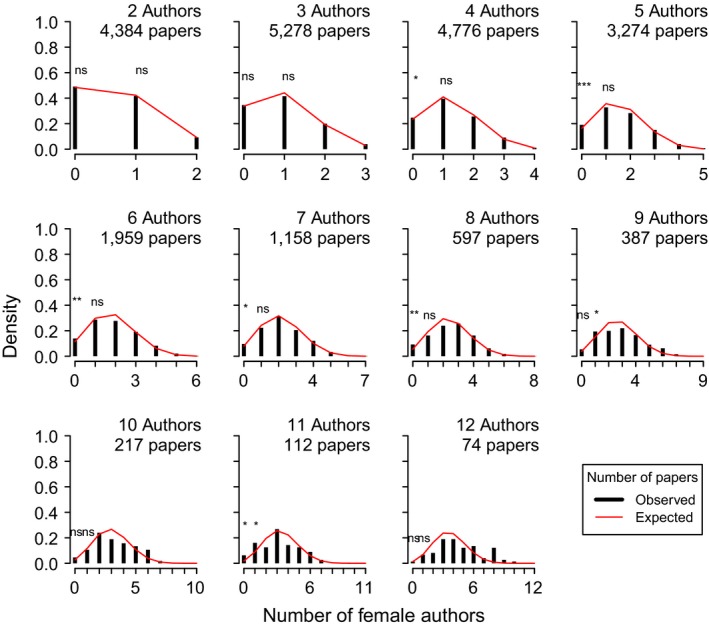
The observed distribution of papers with *N* female authors (black bars) compared to the expected distribution of papers with *N* female authors (red line) for the submitted manuscripts dataset. As in Figure 4, the expectation is derived from a binomial distribution with the same mean as the observed average proportion of female authorship across all author positions, given *N* authors of known gender. There was an overabundance of all‐male manuscripts, in collaborations of four or more authors, but the signal was weaker than in the published papers dataset. There was only minimal support for the “token female” hypothesis, as the number of papers with a single female author did not differ from that expected by the binomial distribution, except in collaborations of 9 and 11 authors

To test the hypothesis that women are more likely to publish with women, and men with men, we tested whether the distribution of genders on multiauthored papers deviated from random expectation. For papers with a given total number of authors, we calculated the expected binomial distribution of the number of female authors given the overall frequency of female authorship. We then compared that expectation against the observed number of female authors with a Kolmogorov–Smirnov test. This analysis ignores author order, but assesses whether the distribution of female authors per paper is as predicted from the overall frequency of women as authors across all papers. Overall, the distribution of the number of female authors on a paper did not deviate from expectations (*p* > 0.05; Figures 4 and A3). This suggests that authorship selection overall is relatively independent of gender. Nevertheless, there was a significant overabundance of all‐male collaborations on papers with three or more authors (Binomial test: *p* < 0.0001). However, there was no evidence that papers included just a single female author (e.g., a “token” female collaborator; Kanter, 2008), as the number of papers with a single female author did not differ from that expected by the binomial distribution for either dataset (*p* > 0.05).

### 3.3 Corresponding authorship

**Figure 5:**
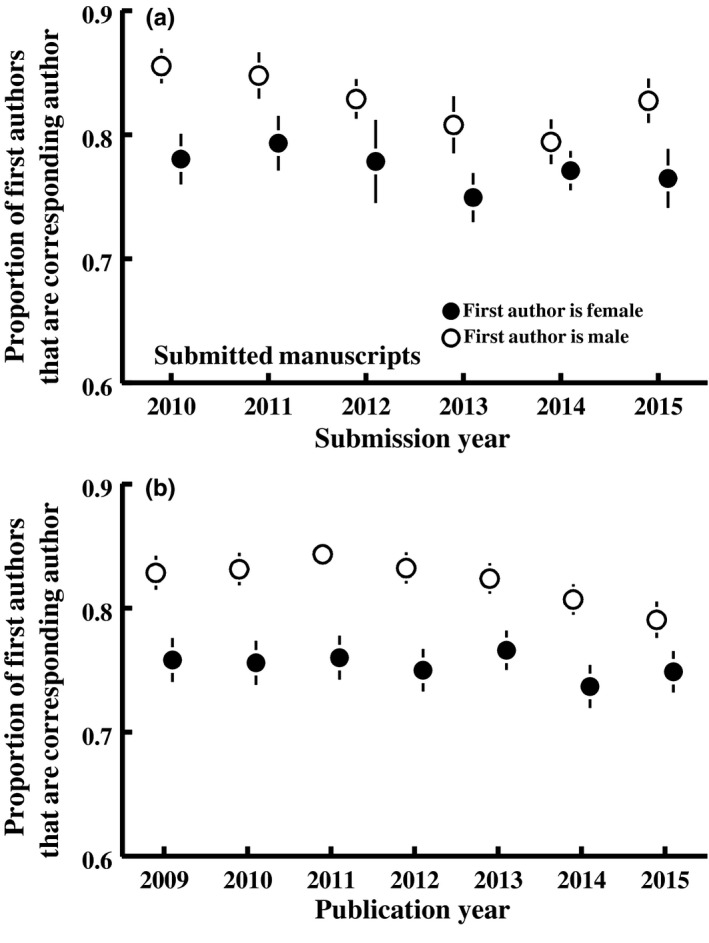
The proportion of first authors that serve as corresponding author for their manuscript differs between male and female first authors.^1^ Means (± *SEM*) were calculated first by averaging across papers within each journal, then across journals within each year. ^1^Analysis (logistic regression): Prob(first author is corresponding author) = *Journal *+ *Year* + *FirstAuthorGender* + 2*‐way interactions. *Submitted papers, panel A: *Year*: χ^2^_1_ = 23.2, *p* < 0.001; *FirstAuthorGender*: χ^2^_1_ = 4.93, *p* = 0.03; Year*FirstAuthorGender: χ^2^_1_ = 4.89, *p* = 0.03; Published papers, panel B: *Year*: χ^2^_1_ = 19.5, *p* < 0.001; *FirstAuthorGender*: χ^2^_1_ = 8.22, *p* = 0.004; Year*FirstAuthorGender: χ^2^_1_ = 8.10, *p* = 0.004

The proportion of women among corresponding authors was less than the proportion of women at the first author position on manuscripts (33.3 ± 0.8 vs. 38.0% ± 0.7% for submitted papers, 31.1 ± 0.8 vs. 35.3 ± 0.5 for published papers; Figure 1). This is because female first authors were less likely to serve as corresponding author of their paper than were male first authors (Figure 5). We found no evidence that the gender of the senior author, or its interaction with the gender of the first author, influenced the likelihood that the first author served as corresponding author of their paper in the submitted papers dataset (add *LastAuthorGender* to the analysis in Figure 5a: χ^2^_1_ = 1.33, *p* = 0.25; LastAuthorGender*FirstAuthorGender: χ^2^_1_ = 0.07, *p* = 0.78). There was some evidence that first authors were more likely to serve as corresponding author when the last author was female in the published papers dataset (add *LastAuthorGender* to the analysis in Figure 5b: χ^2^_1_ = 4.57, *p* = 0.03), though the effect size was very small (only 1%) and there was no significant interaction between the genders of the first and last authors (LastAuthorGender*FirstAuthorGender: χ^2^_1_ = 0.93, *p* = 0.33).

The proportion of corresponding authors that are female increased over time, from 31.5 ± 2.0 to 36.6% ± 2.1% between 2010 and 2015 for submitted papers and 29.1 ± 1.1 to 34.7% ± 0.9% over this same period for the published literature more broadly (Figure 1). This is largely because the proportion of women among first authors is increasing (Figure 1). The gender difference in the probability that a first author served as corresponding author has varied over years, but the proportion of female first authors that serve as corresponding author of their paper has not increased over time (Figure 5). We also found no evidence that the probability the first author served as corresponding author, or that the gender difference in the probability the first author served as corresponding author, varied with journal impact factor (*Impact Factor*: χ^2^_1_ = 1.24, *p* = 0.27; AuthorGender*Impact Factor: χ^2^_1_ = 0.35, *p* = 0.55).

### 3.4 Number of authors

The number of authors on ecology manuscripts has been increasing over time (Fox, Paine, et al., 2016; Gorham & Kelly, 2014; West et al., 2013). 95.2% of submitted manuscripts, and 94.1% of published manuscripts, had more than one author, with the average number of authors on multiauthor papers being 4.37 and 4.29 for the two datasets (averaged across journals and years). The average number of authors on multiauthor papers has gradually but consistently increased between 2010 and 2015, from an average of 4.08 to 4.62 authors per paper in the submitted papers dataset (an increase of 13%; model: *NumberOfAuthors *= Journal* *+ *Year* +Journal*Year; *Year*: *F*_1,22592_ = 144.5, *p* < 0.001) and from 3.90 to 4.55 between 2009 and 2015 in the published papers dataset (an increase of 17%; *F*_1,89807_ = 782.3, *p* < 0.001).

Previous studies have shown that patterns of collaboration differ slightly between men and women; for example, female ecologists generally have fewer unique collaborators over their career than do men (Zeng et al., 2016), though the opposite has been observed in some fields (e.g., industrial–organizational psychologists, Fell & König, 2016). We thus examined whether female first or last authors include fewer coauthors on manuscripts than do male first or last authors. We found the total number of authors to be largely independent of first and last author gender. In the submitted papers dataset, papers with female first or senior authors had, on average across journals and years, just 1.4% and 1.9% more authors, respectively, than did papers with male first or senior authors (of multiauthor papers; adding author gender to the model in the previous paragraph; *F*_1,22592_ = 7.76, *p* = 0.005 and *F*_1,21857_ = 8.68, *p* = 0.003). In the larger published papers dataset, there were no significant differences between male and female first authors in the number of authors on their papers (*F*_1,83145_ = 0.68, *p* = 0.41), and papers with male senior authors had just 4.9% more authors than did papers with female senior authors, a statistically significant (*F*_1,84805_ = 47.5, *p* < 0.001) but generally small difference.

### 3.5 Geographic variation

**Figure 6:**
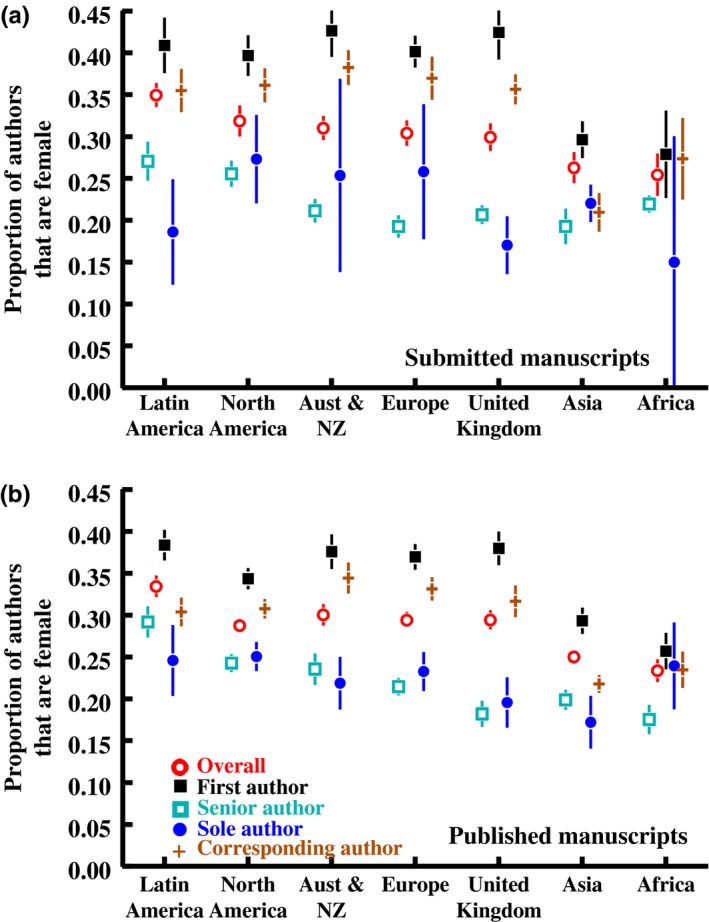
Variation in the proportion of authors that are female^1^ for different positions on the author list among geographic regions for (a) papers submitted to our six focal journals and (b) the published ecology literature. Regions are rank‐ordered (left to right) by the overall proportion of female authors of papers submitted to the six focal journals with first authors from that region in the dataset. Statistical models for panels a^2^ and b^3^ are below. ^1^Means are calculated first by averaged across papers within each year, and then across years within each journal, then across journals within each region. Error bars (standard errors calculated from the among‐year standard deviation) are presented but sometimes smaller than the points. Sample sizes for Oceania (excluding Australia and New Zealand) were very small (*N* = 11 submitted papers and 73 published papers) and so they are excluded from this figure and data analysis. ^2^Variation among geographic regions in the sex ratio of authors of submitted manuscripts: *ProportionFemale *= *Journal* + *Year *+ *Journal* *Year + *GeographicRegion* with *Journal* as a random effect. First author (χ^2^_6_ = 128.9, *p* < 0.001), single author (χ^2^_6_ = 44.5, *p* = 0.075), senior author (χ^2^_6_ = 71.3, *p* < 0.001), corresponding author (χ^2^_6_ = 229.7, *p* < 0.001), overall gender ratio (*F*_6,22217_ = 22.0, *p* < 0.001). ^3^Variation among geographic regions in the sex ratio of authors of published manuscripts: *ProportionFemale *= *Journal* + *Year *+ *GeographicRegion* with *Journal* as a random effect. First author (χ^2^_6_ = 223.1, *p* < 0.001), single author (χ^2^_6_ = 23.8, *p* < 0.001), senior author (χ^2^_6_ = 233.5, *p* < 0.001), corresponding author (χ^2^_6_ = 447.0, *p* < 0.001), overall gender ratio (*F*_6,82020_ = 36.9, *p* < 0.001)

Unsurprisingly, there was substantial variation among the major regions of the world in the proportion of women in the first author position, and the overall proportion of female authors on multiauthor papers (Figure 6). In both datasets, women were better represented as first authors when residing in countries for which gender inequality (*GII*) was lower (adding gender inequality to the model in Figure 1, weighted by the inverse of the number of papers per country*journal*year; *GII*: χ^2^_1_ = 11.9, *p* < 0.001 and χ^2^_1_ = 33.5, *p* < 0.001 for the submitted and published papers datasets, respectively; odds ratios [95% confidence interval], 0.308 [0.158 – 0.602] and 0.494 [0.389 – 0.627], respectively). Similarly, women were better represented as first authors when residing in countries for which women's civil liberties (V‐Dem Women's Civil Liberties Index) are higher (χ^2^_1_ = 11.0, *p* < 0.001 and χ^2^_1_ = 35.3, *p* < 0.001; odds ratios [95% confidence interval], 1.53 [1.34 – 1.76] and 1.60 [1.37 – 1.86], respectively). However, neither gender inequality (*GII*) nor the V‐Dem Women's Civil Liberties Index were adequate to explain all of the variation in the proportion of women as first authors among geographic regions of the world for either dataset (*Author Region* remained a significant predictor when added sequentially after the gender inequality indices; χ^2^_6 _> 22.0, *p* < 0.002 for each analysis).

We observed statistically significant variation among geographic regions in the proportion of first authors that served as corresponding author on their papers (submitted papers: χ^2^_6_ = 1,279.4, *p* < 0.001; published papers: χ^2^_6_ = 4,066.8, *p* < 0.001). On average, first authors were more likely to serve as corresponding author when affiliated with an institution in Australia, New Zealand, and North America, and least likely when from Asia, with other regions of the world intermediate between these. There was some evidence that the difference in the likelihood that male versus female first authors served as corresponding author varied among geographic regions of the world, but this was only statistically significant in the larger published manuscripts dataset (*FirstAuthorGender* added to the model described above; non‐significant FirstAuthorGender*Region interaction for submitted papers: χ^2^_6_ = 11.2, *p* = 0.08; significant interaction for published papers: χ^2^_6_ = 53.9, *p* < 0.001). The likelihood that the first author served as corresponding author was generally higher in more gender equal countries (adding *GII *or *V‐Dem Women Civil Liberties Index*, *WCLI*, to the model in Figure 5, weighted by the inverse of the number of papers per country*journal*year; *GII*: χ^2^_1_ = 24.1, *p* < 0.001 and χ^2^_1_ = 16.3, *p* < 0.001, for submitted and published papers, respectively; *WCLI*: χ^2^_1_ = 7.9, *p* = 0.005 and χ^2^_1_ = 136.3, *p* < 0.001, respectively). Also, in the published papers dataset, the difference between male and female first authors in the likelihood they served as corresponding author increased with increasing inequality (FirstAuthorGender*GII interaction; χ^2^_1_ = 4.74, *p* = 0.03) and with decreasing women's civil liberties (FirstAuthorGender*WCLI interaction; χ^2^_1_ = 14.3, *p* < 0.001); in both cases, female first authors were less likely (compared to male first authors) to serve as corresponding author of their papers when submitting from less gender equal countries. However, neither of these interactions was statistically significant in the smaller submitted papers dataset (χ^2^_1_ = 0.47, *p* = 0.49 and χ^2^_1_ = 0.04, *p* = 0.84).

First authors for whom their country of residence has English as either the most common or an official language were more likely to serve as corresponding authors of their manuscripts (84.4 ± 1.7 vs. 75.8% ± 1.8% for submitted papers, 80.0 ± 1.2 vs. 74.9% ± 1.2% for published papers, averaged across years then journals; χ^2^_1_ = 214.2, *p* < 0.001 and χ^2^_1_ = 580.1, *p* < 0.001, respectively). However, the magnitude of the first author language effect was influenced by the language of the senior author (interaction between first and senior author language; χ^2^_1_ = 28.9, *p* < 0.001 and χ^2^_1_ = 57.8, *p* < 0.001). This analysis is complicated by the fact that most first and senior authors are from countries that are either both English‐speaking or both non‐English‐speaking (90.9% and 92.4% in the two datasets). However, if we limit our comparison to just the 8%–9% of papers for which first and senior authors differ in whether they are from an English‐speaking country, the interaction is clear: the first author serves as corresponding author 88.5% ± 1.1% (submitted papers) or 86.3% ± 0.6% (published papers) of the time when the first author is from an English country and the senior author is not, but just 80.3% ± 1.1% or 78.2 ± 0.6 of the time when the senior author is from an English country and the first author is not (χ^2^_1_ = 21.6, *p* < 0.001 and χ^2^_1_ = 49.1, *p* < 0.001 for submitted and published papers, respectively).

## 4 DISCUSSION

We examined how patterns of authorship differ between men and women in ecology journals. We find that women were less likely to be sole or last author, but more likely to be first author, relative to the overall frequency of female authorship. Women were more likely to be authors on papers with female last authors, but were less well represented on papers published in high impact factor journals. Female first authors were less likely to serve as corresponding author of their papers than were male first authors, and this gender difference increased significantly with the degree of gender inequality in the author's country. First authors from non‐English‐speaking countries were less likely to serve as corresponding author of their papers, especially if the last author was from an English‐speaking country.

### 4.1 Patterns of authorship

Women represent about 30% of all authors in ecology, similar to the figure observed across all fields of science (Larivière et al., 2016). However, women were better represented as first authors on papers in our dataset, albeit only slightly (39% and 35%), than in the global scientific literature (~34%; Larivière et al., 2016). For comparison, the membership of the British Ecological Society, which owns five of the journals in our submitted papers dataset, was 40% women in 2014 (www.britishecologicalsociety.org/making-ecology-for-all-part-2), and the membership of the Ecological Society of America, the comparable North American society, was 37% women as of 2010 (Beck, Boersma, Tysor, & Middendorf, 2014). The representation of women as first authors on papers is thus fairly similar to their representation in these two ecological societies. Across all authorship positions, however, the representation of women is much lower, even when comparing only within the countries that are home to these ecological societies; only 30% of authors from the United Kingdom and 32% of authors from North America are female in the submitted papers dataset (Figure 6a). Women were particularly underrepresented in the last author position, relative to their representation in the community and their representation at other author positions, in both of our datasets (22% and 23%). That women are especially poorly represented among last authors is typical for analyses of authorship in biology and medical journals (e.g., Dotson, 2011; Erren, Groß, Shaw, & Selle, 2014; Feramisco et al., 2009; Jagsi et al., 2006; Holman, Stuart‐Fox, & Hauser, 2018; Kongkiatkamon et al., 2010; West et al., 2013; Wininger et al., 2017). This is likely due to demographic differences between individuals in the various author positions; for example, first authors are commonly students and postdocs, populations for which female representation is quite high in the sciences (Shaw & Stanton, 2012), whereas the last author is commonly the senior scientist for the project, such as the laboratory supervising professor or grant primary investigator (Duffy, 2017; Jagsi et al., 2006), populations in which women remain underrepresented.

Over the past few decades, the proportion of women among authors of papers in ecology has increased substantially (West et al, 2013), a pattern which also holds in our data (Figure 1). We find that the representation of women among both first and last authors has increased consistently over the short time frame (2010–2015) of this study, as has been observed for many fields of study (e.g., Bendals et al., 2017; Chow, Egna, & West, 2017; Fishman, Williams, Goodman, & Ross, 2017; Gu, Almeida, Cohen, Peck, & Merrell, 2017; Helmer, Schottdorf, Neef, & Battaglia, 2017; and references therein), though the proportion of women among last authors remained substantially less than among first authors. These female first authors, who are presumably early in their careers, should gradually transition to the senior author position as they progress and take on research leadership positions. This transition is evident in our data, albeit very subtle; the proportion of women among senior authors significantly increased over the time frame of our study in the published papers dataset. Women were also better represented in our dataset at all positions, including the first and last authorship, than in a similar study of ecology papers archived in JSTOR (West et al., 2013; https://www.eigenfactor.org/gender/#). However, a recent analysis by Holman et al. (2018) suggests that, at current rates of change observed in the biological sciences more broadly, it will be 25–50 years (depending on author position) until women are equally represented at positions other than first author.

Overall, we find that the representation of women among authors did not vary with journal impact factor (Figure 2). This contrasts with previous findings from mathematics (Mihaljević‐Brandt, Santamaría, & Tullney, 2016), biology journals more generally (Bonham & Stefan, 2017), journals in the *Nature Index* (Bendels, Müller, Brueggmann, & Groneberg, 2018), and in biomedical journals (Shen, Webster, Shoda, & Fine, 2018; Strand & Bulik, 2018), all of which showed that women were less well represented as authors in higher impact factor journals (though this pattern was not seen for computational biology journals; Bonham & Stefan, 2017). Those previous studies also generally find that women are less well represented at the more prestigious authorship positions in high impact factor journals than in lower impact factor journals, a pattern we did not observe.

### 4.2 Mixing of genders in multiauthored papers

We find that the proportion of women across all author positions, and at the first author position, is higher when the senior author on a manuscript is female (Figure 3). This generalizes the result observed previously for *Functional Ecology* (Fox, Burns, et al., 2016; Fox, Paine, et al., 2016) across the ecological literature. More generally, papers with only male authors were more abundant than would be expected if collaborations were assembled without consideration of gender, though this pattern was much stronger in the larger published papers dataset (Figure 4) than in the smaller submitted papers dataset (Figure A3). These results are consistent with similar patterns observed in other fields—that women coauthor papers with other women, and men with other men, more often than would be expected if collaborations were assembled without regard for gender (Bonham & Stefan, 2017; Fishman et al., 2017; González‐Alvarez, 2017; Long, Leszczynski, Thompson, Wasan, & Calderwood, 2015; McCann, Ebert, Timmins, & Thompson, 2017; Shah, Huang, Ying, Pietrobon, & O'Brien, 2013). Similar associative gender sorting has been reported for academic mentor–mentee relationships (e.g., Davis, Jacobsen, & Ryan, 2015).

We suggest variation in the proportion of women in different subfields of ecology explains at least some of this variation. Variation in the proportion of women among different subfields of ecology (West et al., 2013) suggests either that research interests differ, albeit slightly, between men and women (Bonnet, Shine, & Lourdais, 2004) or that the proportion of women (relative to men) leaving science varies among subfields. Regardless of the cause, variation in the proportion of women among subfields of ecology will lead to a non‐random association of genders among coauthors, as observed in our data. In addition, or alternatively, women may be more comfortable working with other women, possibly because women tend to both seek and provide more social support than do men in professional environments (Wallace, 2014), and female students tend to feel more belonging, motivation, and confidence when working with female mentors (Dennehy & Dasgupta, 2017). Thus, female graduate students may prefer female mentors (Blake‐Beard, Bayne, Crosby, & Muller, 2011) and female scientists may preferentially collaborate with other women (Jadidi, Karimi, Lietz, & Wagner, 2018). Female scientists may also know more female candidates for mentorships, possibly due to shared experiences or homophily in social networks (Durbin, 2011), and thus be in a better position to scout for female students (Van den Brink & Benschop, 2014). Alternatively, if male scientists preferentially recruit male students and/or collaborators, for reasons other than commonality of research interest, a similar pattern would result, but with more significant implications than the former explanations, given the dominance of men in positions of power in academia (Kern, Kenefic, & Stout, 2015). However, experimental evidence demonstrating discrimination against women in recruitment commonly shows that women express similar degrees of discrimination against female applicants as do men (Milkman, Akinola, & Chugh, 2015; Moss‐Racusin, Dovidio, Brescoll, Graham, & Handelsman, 2012), inconsistent with this latter hypothesis.

### 4.3 Corresponding authorship

Women in ecology journals were more underrepresented as corresponding authors than as first authors on papers because women were less likely than men to assume the role of corresponding author when first author on a paper (Figure 5). This observation was previously reported for the journal *Functional Ecology* (Fox, Burns, et al., 2016; Fox, Paine, et al., 2016) and some biomedical journals (Heckenberg & Druml, 2010; Yun et al., 2015). The analyses we present here show that this pattern holds up across the broader ecological literature. We think the most likely explanation for this difference is that women leave academic research at a higher rate than do men (Fox, 2008; Jadidi et al., 2018; Mihaljević‐Brandt et al., 2016), and thus either defer the final steps of publication to their coauthors or deflect subsequent correspondence (post‐publication queries) to authors who remained in science. Also, women in science typically move between institutions more than do men, for example, as a trailing spouse (Ward & Wolf‐Wendel, 2017), and thus may have less stable contact addresses than do men. Because of this, they may defer correspondence to a coauthor with a more stable contact address. The use of persistent unique identifiers, like Open Researcher and Contributor ID (ORCID), as identifiers of author identity and location, may be helpful in resolving this issue. Alternatively, women may expect to experience gender discrimination in peer review and thus choose to defer to a male collaborator to minimize this discrimination. However, we found no evidence that the gender of the senior author (the most typical alternative corresponding author) predicted how often women defer corresponding authorship. Other alternative hypotheses that we cannot test with our dataset include (a) that women may be less assertive in negotiations with collaborators regarding authorship roles (Amanatullah & Morris, 2010), and thus defer to more senior colleagues more often than do male first authors; (b) that women have less confidence in their roles as lead author, and thus defer the submission process to more confident colleagues; (c) that women have less time available, possibly due to greater responsibilities outside the workplace (Howe‐Walsh & Turnbull, 2016), and thus are more willing (or eager) to defer corresponding authorship to their colleagues; or (d) that women value the corresponding author role less than do men.

Regardless of the reason that women are underrepresented as corresponding author on their paper, deferring corresponding authorship to someone else almost certainly has consequences for a reader's perception of their role in the study. Readers commonly assume that the corresponding author took the lead in the study concept, design, and publication (Bhandari et al., 2004, 2014 ), and perceptions of the first author's role in study concept, design, analysis, and interpretation are significantly reduced when they do not serve as corresponding author on their paper (Bhandari et al., 2003, 2014 ; Wren et al., 2007). Interestingly, the degree to which readers assign primary credit for the research to the corresponding author, rather than the first author, increased with academic rank among medical researchers in China (Jian & Xiaoli, 2013). Though these previous results are for subject areas outside ecology, similar perceptions are likely to be common among ecologists. For example, a survey of ecologists showed that researchers assume that the corresponding author is the person who took responsibility for publishing the manuscript (Duffy, 2017), though that study did not ask whether deferring corresponding authorship to a coauthor reduced the perception of the first author's role in the research. Because women defer corresponding authorships to their coauthors more often than do men, we expect that female first authors have their contributions to their research more commonly undervalued relative to male first authors.

To at least partially counteract the differential perception of contribution, authors should include contribution statements in their manuscripts. Journals should require them (if they do not already do so, Eggerts, 2011) and place them prominently near the author byline, so they can be easily seen by readers. However, author contribution statements alone will not be adequate to address gender biases in perception of research contributions. Author contribution statements only define generalized author roles, which are commonly shared among authors in the first and last author position. Including quantitative estimates of author effort would likely improve the assignment of research credit, but may be impractical to implement. Also, contribution statements commonly declare different roles for men and women first authors, even when sharing the same position in the author byline (Macaluso, Larivière, Sugimoto, & Sugimoto, 2016); these differences could reflect real differences in the roles men and women play in research studies, or differences in the degree to which stated contributions reflect actual contributions (Macaluso et al., 2016). Such gender biases in author‐declarations of effort could possibly extend to any quantitative estimates of effort.

### 4.4 Geographic variation in authorship roles

The proportion of women among authors on papers varied among the major regions of the world; women were better represented as first authors, and more likely to serve as corresponding author on their papers, in more gender equal countries. It seems intuitive that women are more likely to be authors on papers submitted from countries that are more gender equal in rights and opportunities. However, this is counter the observation that women tend to be better represented among STEM graduates in *less* gender equal countries (Stoet & Geary, 2018), possibly because they have fewer career opportunities outside academia in less gender equal societies, and that the representation of women among scientists (across all fields) is uncorrelated among countries with the United Nations gender equality index (Wagner, 2016). One explanation for this inconsistency between geographic patterns in female representation in our authorship data for ecology journals and geographic patterns in STEM and science authorship more generally may be that ecologists (and other life scientists) require lower quantitative skills and, in the United States, require lower quantitative scores on graduate school entrance exams (Ceci, Ginther, Kahn, & Williams, 2014), than do other sciences. It is in mathematics that the gender disparity in performance and anxiety covaries most negatively among countries with gender equality (Stoet, Bailey, Moore, & Geary, 2016), whereas women generally exceed men in reading comprehension, and the degree to which women exceed men in reading comprehension is greatest in more gender equal countries (Stoet & Geary, 2018). These gender differences in math and reading performance (and anxiety) likely contribute to explaining why women are much better represented in the life sciences than in other STEM fields (Ceci et al., 2014; Wagner, 2016), and could explain why we see female representation among authors in ecology increasing with gender equality in their home countries, unlike the patterns observed for STEM programs more broadly.

We also find that first authors were more likely to serve as corresponding author if they reside in a country for which the native language was English, the language in which most (published papers dataset) or all (submitted papers dataset) papers are written. However, the frequency at which authors from non‐native English‐speaking countries serve as corresponding authors varied with the language of their coauthors—first authors deferred to coauthors from English‐speaking countries more often than the reverse. This is unsurprising given that most ecology journals publish in English, and editorial correspondence with authors is typically in English. However, a very large proportion of the English‐speaking authors are in developed countries, whereas authors from non‐English‐speaking countries are a wide mix of high‐ versus low‐income countries. Thus, rather than an influence of language per se, it may be that authors from less scientifically and economically developed communities are deferring the corresponding author role to their colleagues who are more experienced with publishing in international journals (González‐Alcaide, Park, Huamaní, & Ramos, 2017).

### 4.5 Conclusions and a recommendation

Men and women differ in their authorship roles on ecology manuscripts. Women make up just over 30% of all authors on papers in ecology, but less than 25% of last and solo authors. More encouragingly, women make up nearly 40% of first authors, and many of these women will transition to senior authorship roles (including last author) as they progress through their careers, continuing the increase in the representation of women in all authorship positions that has been occurring over the past few decades. However, female first authors delegate or defer corresponding authorship to one of their coauthors more often than do male authors. Given that readers commonly assign substantial credit for research accomplishments to the corresponding author, the greater tendency for women to defer correspondence to a coauthor likely negatively affects the relative amount of credit they receive for their research efforts. This could influence subconscious perceptions of women's contributions to science more generally, or, for those women that stay in academia, could reduce perceptions of their personal contributions and negatively affect their success in academia. We suggest that journals more universally provide a prominent statement of author contributions near the byline of each study. When possible, such contributions should include not just generalized statements of author roles but also specific statements of an author's personal contribution to the study and/or manuscript, including possibly quantitative statements about the magnitude of their contributions, and should declare how and/or why the corresponding author was selected for this role.

## CONFLICT OF INTEREST

The authors have no competing interests.

## AUTHOR CONTRIBUTIONS

CWF and CETP collected and analyzed the data and wrote the manuscript. JPR assisted with data collection and commented on the manuscript.

**Table:** 

	Data collection	Data Analysis	Writing
CWF	40%	60%	90%
CETP	40%	40%	10%
JPR	20%	0%	0%

## DATA ACCESSIBILITY

An anonymized version of the submitted papers dataset, removing personal identifying information (manuscript titles, author names, and author countries), is available on Dryad, https://doi.org/10.5061/dryad.k0h70b0.
